# Self-care behaviours and psychological heath of university staff

**DOI:** 10.1192/j.eurpsy.2025.1991

**Published:** 2025-08-26

**Authors:** A. Abbes, I. Sellami, A. Feki, S. Baklouti, M. L. Masmoudi, M. Hajjaji, K. Jmal Hammami

**Affiliations:** 1 Occupationnal medecine; 2 Rheumatology, CHU Hedi Chaker, Sfax, Tunisia

## Abstract

**Introduction:**

Mindfulness involves being aware of both your thoughts and emotions as well as your surroundings. Regular practice of mindful self-care can enhance the overall well-being. For university staff, these practices can help counteract negative effects on mental health.

**Objectives:**

Our study aims to evaluate the practice of mindful self-care and its impact on the psychological health of university staff.

**Methods:**

We conducted a descriptive, analytical and cross-sectional survey among university staff. The survey was carried out during a one-day training session on mental health promotion using a self-administrated questionnaire. We collected socio-professional data. We assessed psychological health using the depression anxiety and stress scale (DASS 21) and self-care behaviours using Mindful Self-Care Scale (MSCS).

**Results:**

Our study included 65 participants, 67.7% of whom were female. The average age was 53±6.8 years, and the average job tenure was 21.3±8 years. We found that 36.9% of participants experienced mild to moderate stress, 35.4% had mild to moderate depression, 7.7% had severe to extremely severe depression, and 23.1% had severe to extremely severe anxiety.

The average mindful self-care score was 102.8±26.3. The means for specific aspects of mindful self-care were: mindful relaxation 12.6±4.5, physical care 19.9±5.6, self-compassion and purpose 20.7±7.6, supportive relationships 15.4±5.6, supportive structure 13.4±4.6, and mindful awareness 14.5±4.8.

We found a negative correlation between anxiety and depression and mindful self-care (p = 0.02, r = -0.2). In female participants, stress was also negatively correlated with mindful self-care (p = 0.02, r = -0.3).

**Conclusions:**

According to our findings, mindful self-care may be a potential strategy for anxiety, depression and stress management available to university staff. Given the considerably poor psychological health in the study population, we recommend to increase awareness of mindful self-care in university staff.

**Disclosure of Interest:**

None Declared

